# Sustained CREB Phosphorylation Is Associated with Neuritogenic Prostanoid Signaling in NSC-34 Cells

**DOI:** 10.3390/cells15111004

**Published:** 2026-05-29

**Authors:** Koume Nagayama, Hiroshi Nango, Komugi Tsuruta, Hiroko Miyagishi, Yasuhiro Kosuge

**Affiliations:** Laboratory of Pharmacology, School of Pharmacy, Nihon University, 7-7-1 Narashinodai, Funabashi, Chiba 274-8555, Japan; phko23005@g.nihon-u.ac.jp (K.N.); nango.hiroshi@nihon-u.ac.jp (H.N.); phko22004@g.nihon-u.ac.jp (K.T.); miyagishi.hiroko@nihon-u.ac.jp (H.M.)

**Keywords:** NSC-34 cell, prostaglandin, neurite outgrowth, cAMP, protein kinase A, cAMP response element binding protein

## Abstract

**Highlights:**

**What are the main findings?**

- EP2- and IP-mediated prostanoid signaling produced divergent neuritogenic outcomes in NSC-34 cells.
- Sustained CREB phosphorylation, not peak CREB activation, distinguished neuritogenic PGE_2_ signaling from non-neuritogenic PGI_2_ signaling.

**What are the implications of the main findings?**

- Bulk cAMP-PKA activation is insufficient to define neuritogenic Gs-coupled prostanoid signaling.
- Temporal CREB signaling may help explain receptor-specific neuritogenic responses among Gs-coupled prostanoid pathways.

**Abstract:**

Neuritogenesis is essential for neuronal development and circuit formation. Although cAMP signaling downstream of Gs-coupled receptors is considered pro-neuritogenic, activation of these Gs-coupled receptors can produce divergent cellular outcomes. We previously showed that prostaglandin E_2_ (PGE_2_) induces neurite outgrowth in NSC-34 motor neuron-like cells predominantly through Gs-coupled E-prostanoid receptor 2 (EP2) signaling. The I-prostanoid receptor (IP) is also Gs-coupled, but whether its ligand PGI_2_ elicits neuritogenesis remains unclear. Here, we compare the neuritogenic and signaling responses to PGE_2_ and PGI_2_ in NSC-34 cells. PGE_2_ and the EP2 agonist butaprost increased the proportion of neurite-bearing cells, whereas PGI_2_ and the IP agonist beraprost had no effect. PGI_2_ and PGE_2_ induced comparable cAMP accumulation and protein kinase A substrate phosphorylation, and elicited peak cAMP response element binding protein (CREB) phosphorylation at 1 h. However, only PGE_2_ maintained significant CREB phosphorylation at 3–6 h. RNA sequencing at 4 h revealed broadly concordant transcriptional responses, while direct comparison identified Fst as the only gene expressed at higher levels under PGE_2_ than under PGI_2_. These findings suggest that the temporal profile of CREB phosphorylation, rather than the magnitude of early cAMP-PKA signaling, may be associated with differences in neuritogenic outcomes of Gs-coupled prostanoid signaling.

## 1. Introduction

As neurons differentiate, they extend neurites that mature into axons and dendrites; the extent of this outgrowth often correlates with the progression of differentiation [1]. This process is particularly critical for spinal motor neurons, which extend exceptionally long axons from the ventral horn of the spinal cord to innervate target muscles at the neuromuscular junction [2]. Given this unique morphology, motor neurons are vulnerable to defects in axonal outgrowth and maintenance, as exemplified by motor neuron diseases including amyotrophic lateral sclerosis, in which progressive axonal degeneration precedes motor neuron loss [3], and spinal muscular atrophy, which is characterized by developmental defects in motor axon outgrowth [4]. Therefore, elucidating the signaling mechanisms that govern neurite formation and outgrowth is central to understanding motor neuron differentiation and vulnerability.

Among intracellular signaling pathways implicated in neurite outgrowth, cAMP is a well-established pro-neuritogenic second messenger [5,6]. In primary cultured rat motor neurons, pharmacological elevation of intracellular cAMP levels enhances neurite outgrowth, whereas reducing cAMP activity suppresses neurite initiation [7], suggesting that cAMP accumulation is important for neurite outgrowth. The upregulation of cAMP may occur via activation of adenylyl cyclase by Gs-coupled G protein-coupled receptors (GPCRs) [8]. However, cAMP signaling is compartmentalized, enabling different GPCRs to produce distinct downstream outputs even when upregulating cAMP levels [9]. For example, corticotropin-releasing hormone receptor 1-induced cAMP depends on both transmembrane and soluble adenylate cyclase, whereas β-adrenergic receptor-induced cAMP is mediated by transmembrane adenylate cyclase; moreover, corticotropin-releasing hormone receptor 1-driven neurite outgrowth is selectively blocked by inhibition of soluble adenylyl cyclase in mouse hippocampal HT22 cells [10]. cAMP-dependent neuronal differentiation outputs have been reported to diverge at the cAMP sensor level and to mediate distinct effector pathways for neuritogenesis and growth arrest in neuroendocrine NS-1 cells [11]. These observations indicate that the neuritogenic efficacy of Gαs-coupled receptor signaling can be shaped by receptor-specific features of cAMP production and downstream signal routing, rather than solely by the magnitude of bulk cAMP elevation.

Prostaglandins (PGs) are bioactive lipid mediators derived from arachidonic acid that act via distinct prostanoid GPCRs coupled to Gs-, Gi-, and Gq-mediated cascades [12]. NSC-34 cells are a hybrid cell line generated by the fusion of mouse neuroblastoma cells with motor neuron-enriched spinal cord cells and retain motor neuron-like electrophysiological and cholinergic features [13,14]. We previously showed that PGE_2_ promotes neurite outgrowth in NSC-34 cells, an effect inhibited by the E-prostanoid receptor 2 (EP2) antagonist PF-04418948 but not by the EP3 antagonist L-798106, indicating that the neuritogenic response is predominantly mediated by the Gs-coupled EP2 signaling [15]. In contrast, although PGD_2_ induces neurite outgrowth in NSC-34 cells, this effect is not mediated by the Gs-coupled D-prostanoid receptor 1 [16]. These findings suggest that neuritogenic outputs diverge across PGs in NSC-34 cells and are not readily predicted based solely on receptor class or nominal G-protein coupling.

Prostaglandin I_2_ (PGI_2_) is another major PG that predominantly signals through the Gs-coupled I-prostanoid receptor (IP). The PGI_2_ analog iloprost promotes neurite outgrowth in primary cultured mouse cortical neurons, and this effect is abolished by Rp-cAMPS, indicating a cAMP-dependent growth program [17]. Furthermore, IP agonists such as cicaprost and iloprost elicit robust cAMP responses that can exceed those induced by PGE_2_ in primary cultured rat dorsal root ganglia [18]. However, whether PGI_2_/IP signaling induces neurite outgrowth and differentiation of motor neurons remains unclear. In the present study, we investigated the neuritogenic potential of PGI_2_ in NSC-34 cells and compared its downstream signaling responses with those of PGE_2_.

## 2. Materials and Methods

### 2.1. Cell Cultivation and Reagents

NSC-34 cells were provided by Dr. Neil Cashman (University of Toronto, Toronto, ON, Canada) and maintained in Dulbecco’s Modified Eagle Medium (Merck KGaA, Darmstadt, Germany) including 10% fetal bovine serum (Life Technologies Corporation, Carlsbad, CA, USA), 1% penicillin–streptomycin (Life Technologies Corporation) at 37 °C in a humidified 5% CO_2_ incubator. For experiments, undifferentiated cells up to passage 20 were seeded at 1.2 × 10^4^ cells/cm^2^ and treated the following day with PGE_2_ (Tokyo Chemical Industry, Tokyo, Japan), PGI_2_ (FUJIFILM Wako Pure Chemical Corporation, Osaka, Japan), butaprost (Cayman Chemical, Ann Arbor, MI, USA), or beraprost (Merck KGaA). PGI_2_ and beraprost were dissolved in distilled water. PGE_2_ was dissolved in dimethyl sulfoxide (Merck KGaA). Butaprost was dissolved in methyl acetate and ethanol.

### 2.2. Western Blot Analysis

Western blot analysis was performed as previously described [19]. Protein extracts were electrophoresed on 10% sodium dodecyl sulfate (SDS)-polyacrylamide gels and transferred to polyvinylidene difluoride membrane with a pore size of 0.45 µm (Merck KGaA). The membrane was incubated with anti-IP receptor polyclonal antibody (#160070, 1:500, Cayman Chemical), anti-EP2 receptor polyclonal antibody (#101750, 1:1000, Cayman Chemical), anti-EP3 receptor polyclonal antibody (#101760, 1:1000, Cayman Chemical), anti-Phospho-CREB (Ser133) monoclonal antibody (#9198, 1:2000, Cell Signaling Technology, Danvers, MA, USA), anti-CREB1 monoclonal antibody (#67927-1-Ig, 1:10,000, Proteintech, Rosemont, IL, USA) or anti-β-actin antibody (#A5441, 1:2000, Merck KGaA; #PM053-7, 1:4000, Medical & Biological Laboratories, Tokyo, Japan) for 1 h at room temperature with rolling and then overnight at 4 °C as necessary. The membrane was washed three times and incubated with peroxidase-conjugated anti-rabbit antibody (#NA934, Global Life Sciences Technologies Japan K.K., Tokyo, Japan) or anti-mouse antibody (#A28177, Thermo Fisher Scientific, Waltham, MA, USA; #sc-2005, Santa Cruz Biotechnology, Dallas, TX, USA) for 1 h at room temperature. Target protein was detected using ECL Prime (Global Life Sciences Technologies Japan K.K.) or ImmunoStar LD (FUJIFILM Wako Pure Chemical Corporation) and imaged with an Amersham ImageQuant 800 (Global Life Sciences Technologies Japan K.K.). Band intensities were quantified using ImageJ ver. 1.54d (NIH, Bethesda, MD, USA) with the Band/Peak Quantification Tool macro [20].

### 2.3. Immunohistochemistry

Immunohistochemistry was performed as previously described [21]. Adult male B6SJL mice were maintained on a 12 h light/dark cycle at 24 ± 1 °C with ad libitum access to food and water. All experiments involving animals were conducted in accordance with the ethical protocols approved by the Animal Care and Use Committee of Nihon University (Tokyo, Japan; approval no. #AP13P001-1). The experimental unit was an individual animal, and four mice were analyzed for this experiment. Mice were deeply anesthetized and euthanized by an overdose of pentobarbital (100 mg/kg, i.p.), after which lumbar spinal cord tissues were collected for immunohistochemistry. The experimental unit was an individual animal, and four mice were analyzed for this experiment. Because this experiment was designed as a descriptive localization study, no a priori sample size calculation was performed, and no formal inclusion or exclusion criteria were prespecified. Therefore, no animals or sections were excluded from the analysis. The free-floating sections of mouse lumbar spinal cord were incubated with a primary antibody against IP (#160070 1:500, Cayman Chemical) for 72 h at 4 °C. After washing with PBS, the sections were incubated for 2 h with Cy5-conjugated secondary antibody (1:500; Jackson ImmunoResearch Laboratories, West Grove, PA, USA) and stained with NeuroTrace Fluorescent Nissl Stains (Life Technologies Corporation). The sections were analyzed using a confocal laser microscope (LSM-710, Zeiss, Oberkochen, Germany). Motor neurons were defined as follows: (i) Nissl stain-positive; (ii) localization in ventral horns; (iii) diameter > 25 μm.

### 2.4. 3-(4,5-Dimethyl-2-thiazolyl)-2,5-diphenyl-2H-tetrazolium Bromide (MTT) Assay

To assess the cell proliferation capability, MTT assays were performed as previously described [15]. Briefly, cells were incubated with 0.5 mg/mL MTT for 3 h at 37 °C. The resulting formazan was solubilized in 20% SDS and 50% N,N-dimethylformamide solution (pH 4.7). After overnight incubation, absorbance was measured at a 570 nm test wavelength and 655 nm reference wavelength using a microplate reader (SH-1000Lab, Corona Electric, Ibaraki, Japan).

### 2.5. Neurite Outgrowth Analysis

Neurite outgrowth was analyzed following our previous procedure [15,22]. Briefly, NSC-34 cells were photographed using phase-contrast microscopy (IX70, Olympus, Tokyo, Japan) with an i-NTER LENS (Micronet, Saitama, Japan) after treatment with each reagent for 48 h. For each condition, 50 cells/well were randomly selected in each independent experiment. For quantification of the neurite-bearing cell percentage, images were coded by an independent laboratory member, and neurite outgrowth was assessed by an observer blinded to the treatment conditions. Neurite-bearing cells were defined as cells with neurites longer than one cell-body diameter, and the percentage of neurite-bearing cells was calculated.

### 2.6. Measurement of cAMP Levels

Intracellular cAMP levels were measured using a cAMP-Glo Assay kit (#V1501, Promega K.K., Tokyo, Japan) according to the manufacturer’s instructions. NSC-34 cells were seeded in white 96-well plates with clear bottoms and incubated overnight at 37 °C in 5% CO_2_. Cells were treated with PGE_2_ or PGI_2_ for either 10 min or 3 h. For the 10 min condition, cAMP quantification was performed immediately after treatment. For the 3 h condition, cells were first treated with PGE_2_ or PGI_2_ for 3 h, after which phosphodiesterase inhibitors (3-isobutyl-1-methylxanthine, 500 μM; Ro 20-1724, 100 μM) were added to prevent cAMP degradation. Luminescence was measured using a microplate reader (FLUOstar Omega, BMG Labtech, Ortenberg, Germany).

### 2.7. RNA Sequencing and Differential Expression Analysis

Undifferentiated NSC-34 cells were treated with PGE_2_ (30 µM), PGI_2_ (30 µM), or a control for 4 h. Total RNA was extracted using a High Pure RNA Isolation Kit (Roche, Basel, Switzerland), and samples were submitted to Rhelixa (Tokyo, Japan) for quality control, library preparation, and RNA sequencing. RNA concentration and quality were assessed by electrophoretic analysis prior to library preparation. Poly(A)+ RNA was isolated using the NEBNext Poly(A) mRNA Magnetic Isolation Module (New England Biolabs, Ipswich, MA, USA), and strand-specific libraries were prepared using a NEBNext Ultra II Directional RNA Library Prep Kit (New England Biolabs). Libraries were sequenced on an Illumina NovaSeq X Plus platform to generate 150 bp paired-end reads (PE150), yielding ~6 Gb/sample (~40 million reads/sample; ~20 million read pairs). Raw reads were quality-checked using fastp ver. 1.0.1 [23], and adapter/low-quality trimming was performed when necessary. Reads were aligned to the mouse reference genome GRCm39 using STAR ver. 2.7.11b [24]. Gene-level counts were generated using featureCounts with GENCODE vM38 annotation. Differential expression analysis was performed using DESeq2 ver. 1.50.2 [25] in R ver. 4.5.2 (The R Foundation, Vienna, Austria). Counts were normalized using DESeq2 size factors, and shrunk log_2_ fold changes (log2FC) were estimated using apeglm. For the PGE_2_/control (Ctrl) vs. PGI_2_/Ctrl concordance plot, per-gene shrunk log_2_FC values were plotted, and Pearson’s r, Spearman’s rho, and linear regression were calculated. Gene set enrichment analysis was performed on pre-ranked gene lists using a signed gene-level statistic derived from the DESeq2 differential expression analysis. Gene sets were based on Gene Ontology (GO) biological process, molecular function, cellular component, and Kyoto Encyclopedia of Genes and Genomes (KEGG) terms, and enrichment significance was assessed by permutation with multiple-testing correction (FDR).

### 2.8. Statistical Analysis

All data are presented as mean ± standard deviation (SD) or standard error of the mean (SEM). Statistical analyses were performed using R ver. 4.5.2 and GraphPad Prism ver. 11.0 (GraphPad Software, Boston, MA, USA). Normality of data distribution was assessed using the Shapiro–Wilk test before the application of parametric tests. The choice of parametric or nonparametric tests was determined independently for each dataset based on the results of normality testing. For comparisons among three or more groups, one-way ANOVA was used, followed by Tukey’s post hoc multiple-comparisons test for all pairwise comparisons or Dunnett’s post hoc test for comparisons with a single control. When the assumption of normality was not satisfied, the Kruskal–Wallis test followed by Dunn’s post hoc test was applied. Statistical significance was set at *p* < 0.05. For RNA sequencing, *p*-values were adjusted using the Benjamini–Hochberg method. Genes with an adjusted *p* (*p*-adj) ≤ 0.05 and |log_2_fold change (FC)| ≥ 1 were considered differentially expressed. No statistical analysis was performed for the spinal cord immunohistochemistry, as this experiment was conducted as a descriptive localization study.

## 3. Results

### 3.1. Undifferentiated NSC-34 Cells Express EP2, EP3, and IP

**Figure 1:**
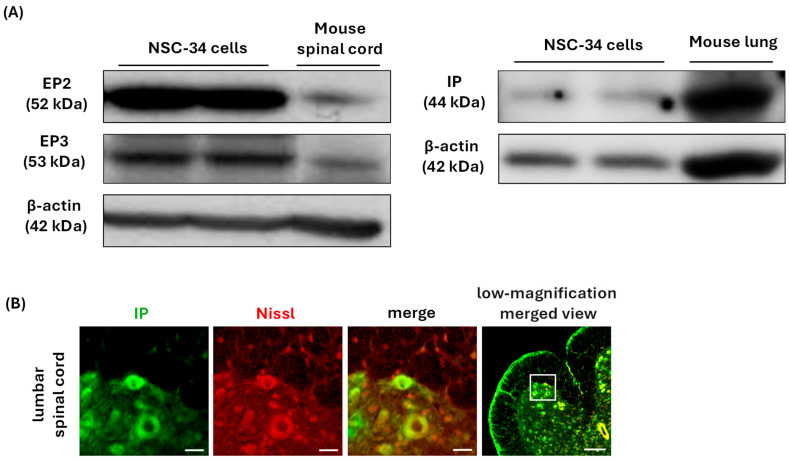
Expression of EP and IP receptors in NSC-34 cells. (**A**) Representative bands of EP receptors (EP2 and EP3) and IP receptors in undifferentiated NSC-34 cells. Mouse spinal cord and lung lysate were used as positive controls. β-Actin was used as a loading control. Representative data from four biologically independent experiments are shown. (**B**) Representative confocal images of the ventral horn region of lumbar spinal cord sections from 18-week-old mice. Sections were immunostained for the IP receptor (green) and counterstained with NeuroTrace fluorescent Nissl stain (red). Yellow signals indicate overlap between IP-like immunoreactivity and Nissl staining. The white boxed area in the low-magnification merged view is shown at higher magnification in the left panels. Representative data from four biologically independent experiments are shown. Scale bar: 20 μm for the high-magnification images and 100 µm for the low-magnification image.

We first verified the expression of prostanoid receptors in undifferentiated NSC-34 cells by Western blotting. Consistent with previous reports [26], EP2 and EP3 were detected, and the corresponding bands were also observed in the mouse spinal cord used as a positive control. IP receptor was observed in NSC-34 cells, with a corresponding band confirmed in mouse lung lysate [27] (Figure 1). To investigate the localization of IP in motor neurons, we performed fluorescent double-immunostaining of IP using NeuroTrace Nissl. Confocal microscopy showed IP-like immunoreactivity in the ventral spinal cord, including Nissl-positive motor neuron-like cells (Figure 1). Together with the Western blotting data, these observations support the presence of the receptor subtypes required for experimental comparisons in NSC-34 cells.

### 3.2. PGI_2_ and the IP Agonist Beraprost Do Not Affect MTT Reduction

**Figure 2:**
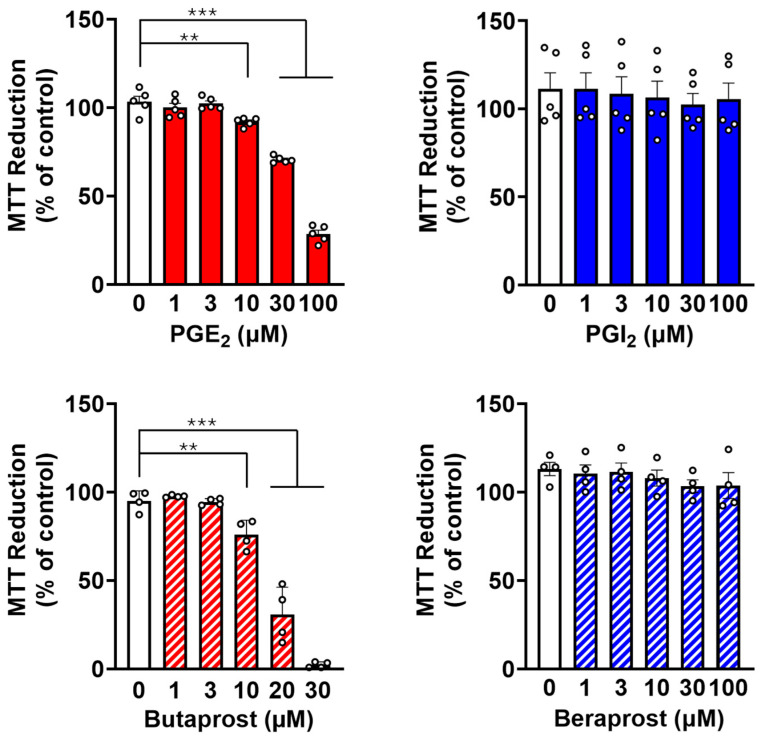
Effects of prostaglandin (PG)-E_2_, PGI_2_, butaprost, and beraprost on MTT reduction activity. Undifferentiated NSC-34 cells were treated with PGE_2_, PGI_2_, butaprost, or beraprost for 48 h at the indicated concentrations. Graphs show the concentration-dependent effects of PGE_2_, PGI_2_, butaprost, and beraprost on MTT reduction activity. The results are expressed as a percentage relative to the non-treated cells. Values represent mean ± SEM from 4–5 biologically independent experiments. Statistical significance was determined using one-way ANOVA followed by Dunnett’s post hoc test. ** *p* < 0.01, *** *p* < 0.001.

To evaluate the effects of PGI_2_ on MTT reduction activity, we compared MTT reduction in undifferentiated NSC-34 cells treated with PGE_2_ or PGI_2_ (Figure 2). Consistent with our previous report [15], treatment with PGE_2_ (1–100 μM) for 48 h decreased MTT reduction activity in a concentration-dependent manner, with significant decreases at concentrations of 10 µM and above. Similarly, butaprost, a selective EP2 agonist, reduced MTT reduction activity in a concentration-dependent manner at concentrations of 1–30 μM, with significant decreases at 10 μM and above. In contrast, PGI_2_ (1–100 μM) showed no detectable effect.

To determine whether this lack of effect reflects insufficient IP receptor engagement or ligand stability, we used beraprost, a stable and selective IP receptor agonist. Similar to PGI_2_, beraprost (1–100 μM) did not alter MTT reduction. These results indicate that, unlike PGE_2_ and the EP2 agonist butaprost, both PGI_2_ treatment and pharmacological activation of the IP receptor do not affect MTT reduction activity in undifferentiated NSC-34 cells.

### 3.3. PGI_2_ and the IP Agonist Beraprost Do Not Affect Neurite Outgrowth

**Figure 3:**
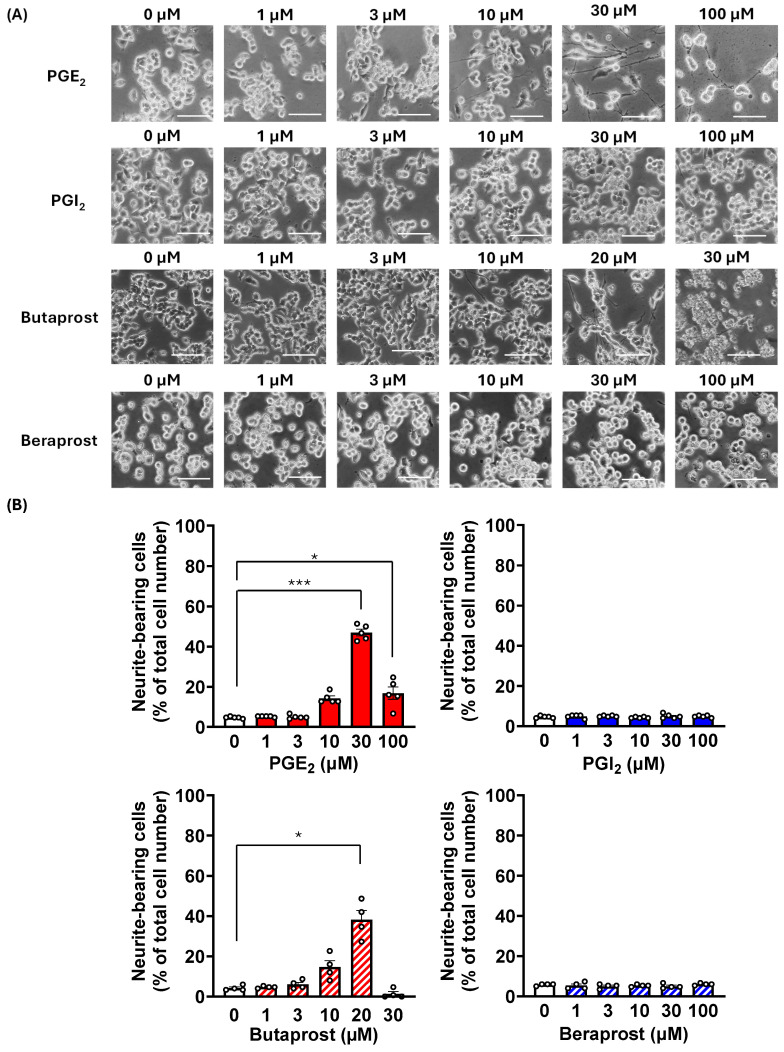
Effects of PGE_2_, PGI_2_, butaprost, and beraprost on neurite outgrowth. Undifferentiated NSC-34 cells were treated with PGE_2_, PGI_2_, butaprost, or beraprost for 48 h at the indicated concentrations. (**A**) Representative phase-contrast images of NSC-34 cells from each experimental group. Scale bar: 100 µm. (**B**) Concentration-dependent effects of PGE_2_, PGI_2_, butaprost, and beraprost on neurite outgrowth, measured as the percentage of cells extending neurites. Neurite quantification was performed in a blinded manner. Neurite-bearing cells were scored according to predefined criteria under identical imaging conditions. Values represent mean ± SEM from 4–5 biologically independent experiments. Statistical significance was determined using the Kruskal–Wallis test followed by Dunn’s post hoc test. * *p* < 0.05, *** *p* < 0.001.

We assessed whether PGI_2_ and beraprost induce neurite outgrowth in NSC-34 cells by measuring the percentage of neurite-bearing cells (Figure 3). In the vehicle-treated group, most cells displayed a rounded morphology with few visible neurites (Figure 3A). As reported previously [15], PGE_2_ increased the proportion of neurite-bearing cells in a concentration-dependent manner, reaching significance at concentrations of ≥10 µM (Figure 3B). The maximal response was observed at 30 µM PGE_2_, where neurite outgrowth was detected in 46.9% of cells (Figure 3B). Similarly, butaprost (1–30 μM) increased the proportion of neurite-bearing cells in a concentration-dependent manner, reaching significance at concentrations of ≥10 μM. The maximal response was observed at 20 μM butaprost, with neurite outgrowth detected in 38.2% of cells (Figure 3B). In contrast, neither PGI_2_ nor beraprost (1–100 μM) increased the proportion of neurite-bearing cells, and values remained comparable to vehicle-treated cells (Figure 3B). These findings indicate that IP receptor stimulation is insufficient to induce neurite outgrowth in NSC-34 cells.

### 3.4. PGE_2_ and PGI_2_ Induce Comparable Early Bulk cAMP Production and PKA Substrate Phosphorylation

**Figure 4:**
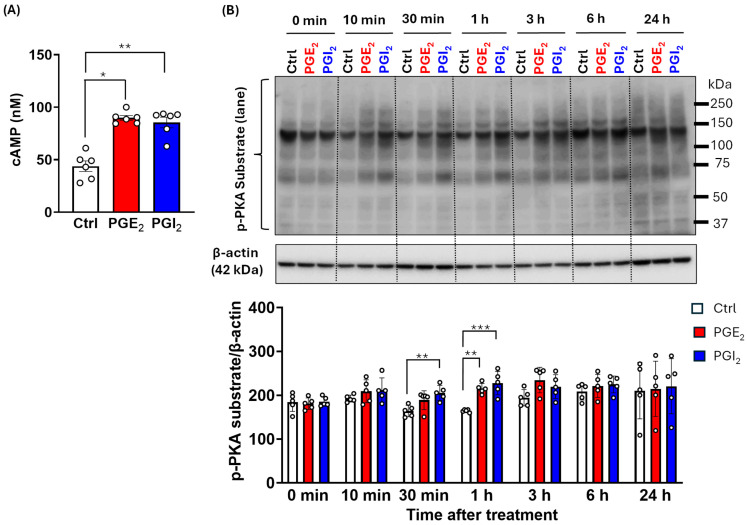
Effects of PGE_2_ and PGI_2_ on early cAMP accumulation and phosphorylation of protein kinase A (PKA) substrate proteins. Undifferentiated NSC-34 cells were treated with PGE_2_ (30 µM) or PGI_2_ (30 µM). (**A**) Intracellular cAMP accumulation during 10 min stimulation. Data are presented as mean ± SEM from six biologically independent experiments. Statistical significance was determined using the Kruskal–Wallis test followed by Dunn’s post hoc test. * *p* < 0.05, ** *p* < 0.01. (**B**) Cells were treated with PGE_2_ (30 µM), PGI_2_ (30 µM), or medium-only control (Ctrl) for the indicated times, and phosphorylation of PKA substrate proteins (p-PKA substrates) was analyzed using Western blotting. Photographs show representative bands of p-PKA substrates, with β-actin as an internal control. The graph shows densitometric quantification of total p-PKA substrate signals normalized to the corresponding β-actin levels. Data are presented as mean ± SD from five biologically independent experiments. Statistical significance was determined using one-way ANOVA followed by Tukey’s post hoc test. ** *p* < 0.01, *** *p* < 0.001.

Since EP2 and IP are both Gs-coupled receptors [28], we examined whether IP receptor signaling engages the Gs-cAMP-PKA pathway in NSC-34 cells. Based on the concentration-response analysis for neurite outgrowth (Figure 3), 30 μM was selected as the concentration at which PGE_2_ produced a maximal neuritogenic response, and PGI_2_ was tested at the same concentration to allow direct comparison of downstream signaling. Both PGE_2_ (30 μM) and PGI_2_ (30 μM) significantly increased intracellular cAMP levels within 10 min compared with the vehicle (Figure 4A). These results indicate that PGI_2_ elicited an early bulk cAMP response comparable to that of PGE_2_, despite failing to induce neurite outgrowth.

To determine whether this similarity extends to the immediate downstream effector PKA, we assessed the phosphorylation of PKA substrate proteins at 0, 10, and 30 min and 1, 3, 6, and 24 h. Stimulation with PGI_2_ significantly increased the phosphorylation of PKA substrate proteins at 30 min. Both PGE_2_ and PGI_2_ elicited significant increases in this phosphorylation at 1 h compared with their respective time-matched controls. No significant differences were observed in either the time course or magnitude of PKA substrate phosphorylation between PGE_2_ and PGI_2_ (Figure 4B). Collectively, these findings support the functional coupling of IP to the Gs-cAMP-PKA signaling cascade in NSC-34 cells.

### 3.5. PGE_2_ Sustains CREB Phosphorylation More than PGI_2_

Although PGE_2_ and PGI_2_ produced comparable early cAMP accumulation and PKA substrate phosphorylation, their neurite-outgrowth phenotypes diverged. Therefore, we examined the activation of CREB, a transcription factor implicated in neurite outgrowth and a downstream target of the Gs-cAMP pathway, at 0, 10, and 30 min and 1, 3, 6, and 24 h after stimulation [quantified as the ratio of phosphorylated CREB (p-CREB) to total CREB].

**Figure 5:**
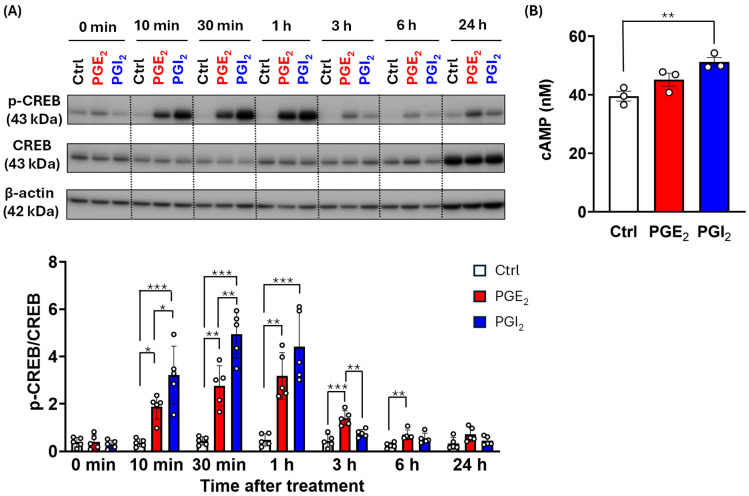
Effects of PGE_2_ and PGI_2_ on phosphorylation of cAMP response element-binding protein (CREB) and persistence of intracellular cAMP elevation. Undifferentiated NSC-34 cells were treated with PGE_2_ (30 µM) or PGI_2_ (30 µM). (**A**) Cells were treated with PGE_2_ (30 µM), PGI_2_ (30 µM), or medium-only control for the indicated times, and phosphorylated CREB (p-CREB) and total CREB were analyzed using Western blotting. Photographs show representative bands of p-CREB and total CREB, with β-actin as an internal control. The graph shows densitometric quantification of CREB phosphorylation expressed as the p-CREB/total CREB ratio. Data are presented as mean ± SD from five biologically independent experiments. Statistical significance was determined using one-way ANOVA followed by Tukey’s post hoc test. * *p* < 0.05, ** *p* < 0.01, *** *p* < 0.001 (**B**) Intracellular cAMP levels measured at 3 h after stimulation. Data are presented as mean ± SD from three biologically independent experiments. Statistical significance was determined using one-way ANOVA followed by Tukey’s post hoc test. ** *p* < 0.01.

Stimulation with either PGE_2_ or PGI_2_ rapidly increased the p-CREB/total CREB ratio, reaching statistical significance as early as 10 min, each relative to its time-matched control (Figure 5A). In both groups, the p-CREB/total CREB ratio peaked at 1 h and then declined (Figure 5A). The ratio was significantly higher in PGI_2_-treated cells than that in PGE_2_-treated cells at 10–30 min. However, significant increases persisted at 3 and 6 h only in the PGE_2_-treated group relative to the corresponding control. Furthermore, at 3 h, the p-CREB/total CREB ratio was significantly higher in the PGE_2_ group than in the PGI_2_ group, at which point PGI_2_-induced p-CREB levels returned to baseline (Figure 5A).

To investigate whether this differential persistence of p-CREB reflects differences in sustained cAMP accumulation, we measured intracellular cAMP levels at 3 h after stimulation with PGE_2_ or PGI_2_. Notably, PGI_2_ significantly increased cAMP levels compared with the control group, whereas PGE_2_ did not show a significant increase. Intracellular cAMP levels did not differ significantly between PGE_2_ and PGI_2_ (Figure 5B). These findings indicate that PGE_2_ elicits more sustained CREB phosphorylation than PGI_2_, which is not explained by differences in whole-cell cAMP abundance at the corresponding time point.

### 3.6. Broadly Concordant Early Transcriptional Responses to PGE_2_ and PGI_2_, with Higher Induction of Follistatin (Fst) by PGE_2_

**Figure 6:**
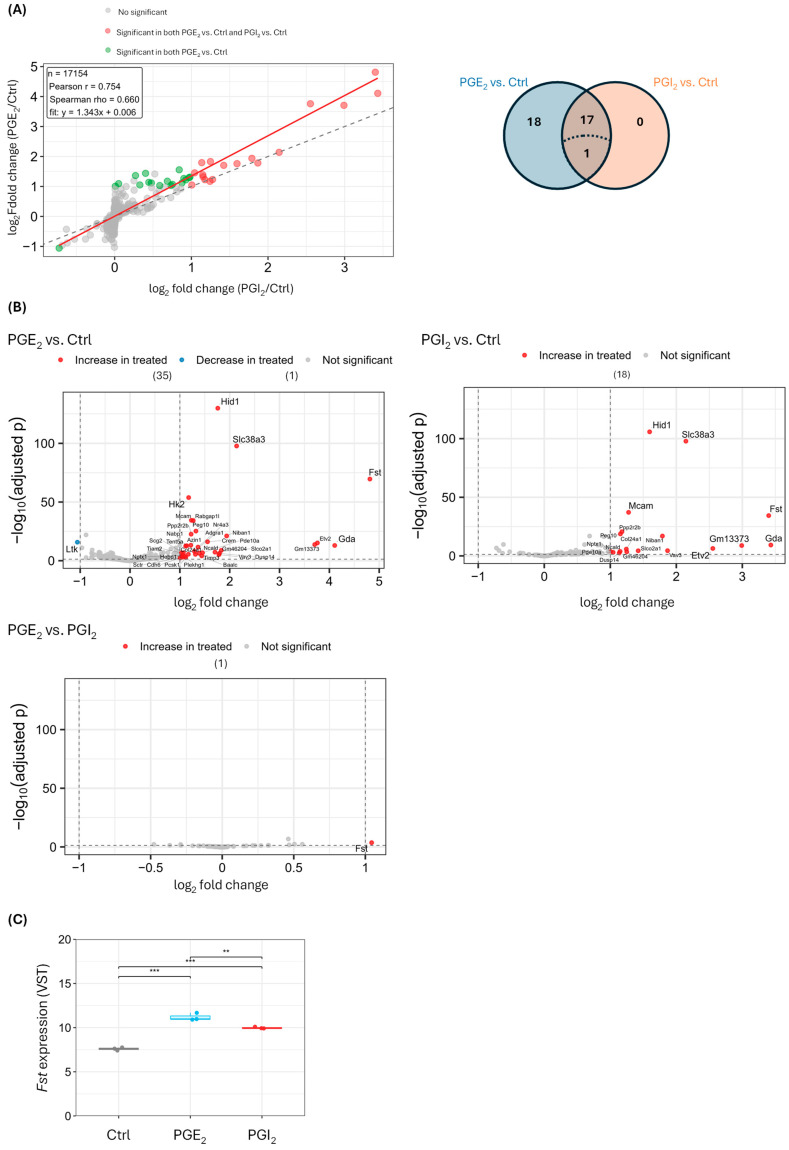

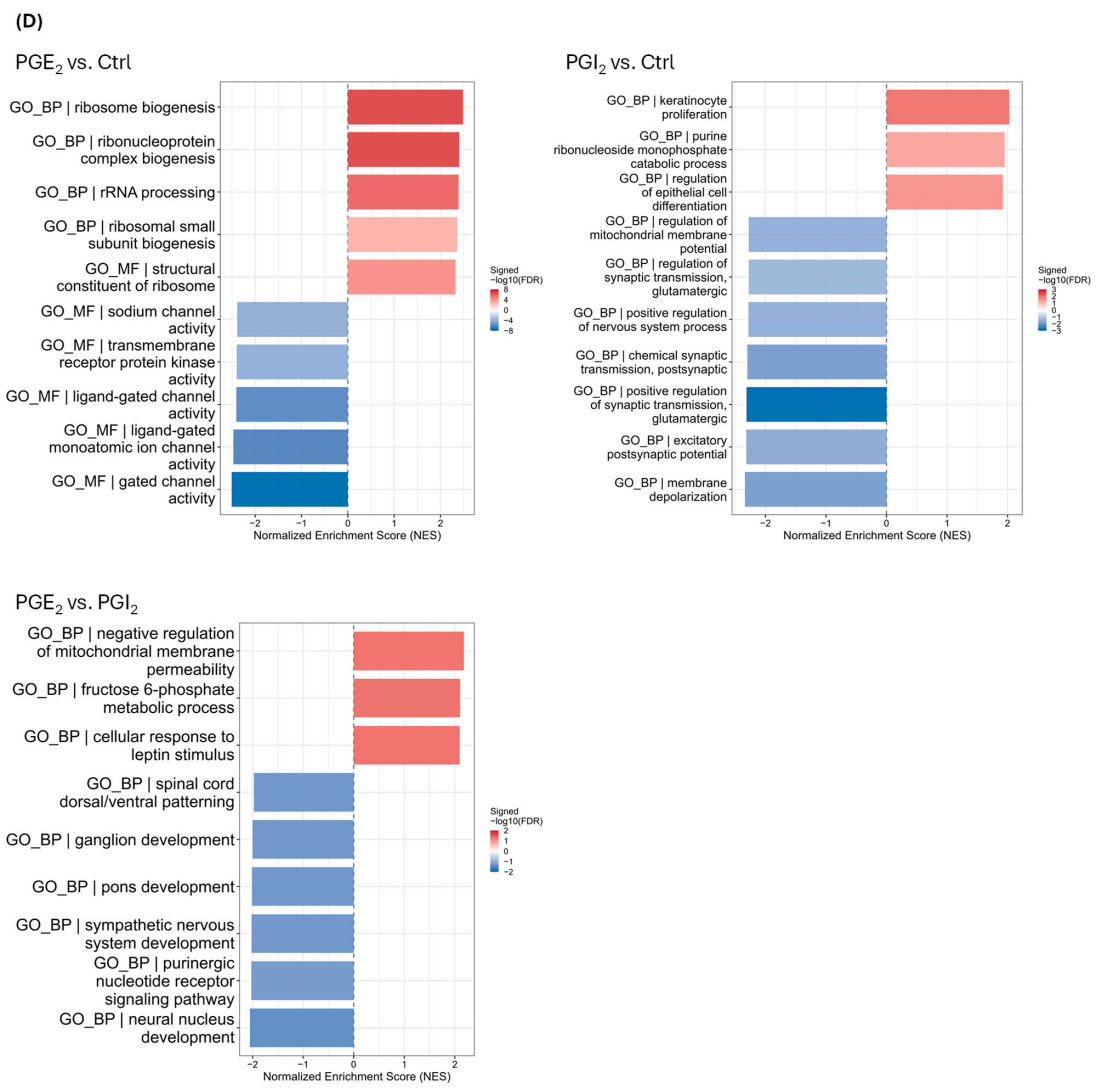
Transcriptomic responses to PGE_2_ and PGI_2_. Undifferentiated NSC-34 cells were treated with PGE_2_ (30 µM), PGI_2_ (30 µM), or medium-only control for 4 h, followed by RNA sequencing, with three biologically independent replicates. (**A**) Concordance plot of gene-wise shrunken log_2_FC for PGI_2_/Ctrl (*x*-axis) vs. PGE_2_/Ctrl (*y*-axis). The dashed line indicates *y* = *x* (perfect agreement), and the red line indicates the linear regression fit. Gray points indicate genes not meeting the significance criteria in either comparison; red points indicate genes significantly upregulated in both PGE_2_ vs. Ctrl and PGI_2_ vs. Ctrl; green points indicate genes significantly upregulated only in PGE_2_ vs. Ctrl (*p*-adj ≤ 0.05 and |log_2_fold-change (FC)| ≥ 1). Venn diagram shows the overlap of differentially expressed genes (*p*-adj ≤ 0.05 and log_2_FC ≥ 1) among PGE_2_ vs. Ctrl, PGI_2_ vs. Ctrl, and PGE_2_ vs. PGI_2_. (**B**) Volcano plots show PGE_2_ vs. Ctrl, PGI_2_ vs. Ctrl, and PGE_2_ vs. PGI_2_ at 4 h. Red and blue points indicate upregulated and downregulated genes, respectively (*p*-adj ≤ 0.05 and |log_2_FC| ≥ 1); gray points are not significant. (**C**) Box-and-jitter plot shows *Fst* expression across Ctrl, PGE_2_, and PGI_2_ using variance-stabilized counts. Statistical significance was determined using one-way ANOVA followed by Tukey’s post hoc test. ** *p* < 0.01, *** *p* < 0.001. (**D**) Graphs show bar plots of normalized enrichment scores from Gene Set Enrichment Analysis for PGE_2_ vs. Ctrl, PGI_2_ vs. Ctrl, and PGE_2_ vs. PGI_2_ at 4 h. Gene Set Enrichment Analysis was performed using Gene Ontology biological process (GO-BP), molecular function (GO-MF), cellular component (GO-CC), and KEGG gene sets. The top 10 terms ranked by NES across all gene sets are shown.

To characterize early transcriptional responses downstream of PG stimulation, we performed RNA sequencing 4 h after treatment with PGE_2_ or PGI_2_. Across 17,769 genes, the log_2_FC relative to control was positively correlated between PGE_2_- and PGI_2_-treated cells (Pearson *r* = 0.716, Spearman *ρ* = 0.655), indicating broadly concordant transcriptional responses at this time point (Figure 6A). Using *p*-adj ≤ 0.05 and |log_2_FC| ≥ 1 as thresholds, 36 genes were significantly changed relative to the control. Of these, 18 were uniquely regulated by PGE_2_, whereas the remaining 18 were commonly regulated by both PGE_2_ and PGI_2_; no genes were uniquely regulated by PGI_2_ (Figure 6A). Notably, one of the genes commonly regulated by both agonists also exhibited differential expression between PGE_2_- and PGI_2_-treated cells (Figure 6A).

Differential expression analysis identified 36 genes in PGE_2_ vs. control, with 35 upregulated and 1 downregulated, and 18 genes in PGI_2_ vs. control, all of which were upregulated (Figure 6B). A complete list of these differentially expressed genes, including fold change values and adjusted p-values, is provided in Supplementary Table S1. Direct comparison of PGE_2_- and PGI_2_-treated cells identified a single gene expressed at higher levels under PGE_2_ than under PGI_2_ (Figure 6B), namely *Fst*, which was upregulated by both agonists relative to the control but reached significantly higher expression under PGE_2_ (Figure 6C).

Gene set enrichment analysis of ranked gene lists suggested differences in pathway-level patterns, including enrichment of RNA-processing/ribosome-related terms in PGE_2_ vs. control and negative enrichment of synaptic signaling-related terms in PGI_2_ vs. control (Figure 6D).

## 4. Discussion

Neuritogenesis represents a key morphogenetic process in neuronal differentiation, wherein the elaboration of primordial neurites establishes the structural blueprint for future axonal and dendritic compartments [29,30]. While these processes are conventionally viewed as canonical downstream consequences of conserved signaling cascades, the present study demonstrates that equivalent activation of the Gs-cAMP-PKA pathway does not necessarily promote neuritogenesis in motor neuron-like NSC-34 cells. Despite PGE_2_ and PGI_2_ eliciting comparable cAMP accumulation and PKA substrate phosphorylation, only PGE_2_ effectively drove neurite outgrowth. This dissociation indicates that the magnitude of bulk second-messenger signaling alone is insufficient to account for neuritogenic outcomes and suggests that receptor-specific features of downstream signaling may influence the cellular response.

In our previous study, EP2 and EP3 were clearly detected in both mouse spinal cord and NSC-34 cells, whereas EP1 and EP4 were not detected [26]. Consistent with this receptor profile, we confirmed that undifferentiated NSC-34 cells express EP2 and EP3 receptors. Because EP2 couples to Gs and EP3 couples to Gi [28], the cellular response to exogenous PGE_2_ should be regarded as the net effect of simultaneous activation of receptors with opposing influences on cAMP signaling. However, in our previous study, the EP2 antagonist PF-04418948 inhibited PGE_2_-induced neurite outgrowth, whereas the EP3 antagonist L-798106 had no significant effect [15], supporting a predominant contribution of EP2 signaling under the present experimental conditions. Although IP receptor expression has been reported in the spinal cord [31], the specific cell types have remained unclear. The present study not only demonstrated IP-like immunoreactivity in Nissl-positive motor neuron-like cells but also identified its expression in NSC-34 cells. Importantly, the presence of both EP2 and IP receptors enabled direct comparison of two Gs-coupled prostanoid pathways within the same cellular context. Collectively, the prostanoid receptor profile observed in NSC-34 cells supports their use as a model for prostanoid signaling in motor neuron differentiation.

Neurite outgrowth is typically coupled with cell-cycle withdrawal during neuronal differentiation [32]. We previously reported that PGE_2_ increases the percentage of neurite-bearing cells while reducing MTT reduction activity without overt cell death in NSC-34 cells [15]. In the present study, PGE_2_ similarly increased the neurite-bearing fraction and reduced MTT reduction activity. At concentrations up to 30 μM, no overt cell death was observed by phase-contrast microscopy, and the decrease in MTT reduction activity at these concentrations is consistent with cell-cycle withdrawal associated with neuronal differentiation. At 100 μM, however, phase-contrast images showed apparent cell death, indicating that the reduction in MTT reduction activity at this concentration reflects, at least in part, cytotoxicity. The selective EP2 agonist butaprost reproduced both effects, providing additional pharmacological support for the predominant contribution of EP2 signaling. Because cytotoxicity-specific assays, such as LDH release, were not performed in the present study, decreases in MTT reduction activity should be interpreted cautiously. Nevertheless, together with the antagonist data [15], these findings support that the neuritogenic and anti-proliferative responses to PGE_2_ are predominantly associated with EP2 signaling. Like EP2, IP is a Gs-coupled receptor that promotes intracellular cAMP accumulation and subsequent activation of PKA-dependent signaling [18]. Although intracellular cAMP accumulation is considered a hallmark of Gs-coupled GPCR activation and a critical mediator of neurite outgrowth in various neurons, including motor neurons [15,33], PGI_2_ increased intracellular cAMP comparable to the effects of PGE_2_ in NSC-34 cells. Moreover, PKA substrate phosphorylation occurred with similar kinetics and magnitude following stimulation with either PGE_2_ or PGI_2_. However, PGI_2_ and beraprost failed to induce neuritogenesis or modulate MTT reduction activity. These results suggest the functional coupling of IP to the Gs-cAMP-PKA signaling cascade in NSC-34 cells, and further indicate that equivalent activation of the Gs-cAMP-PKA pathway does not necessarily result in neuritogenic outcomes.

cAMP-PKA signaling is compartmentalized within cells, and downstream outputs depend not only on signal magnitude but also on the spatial and temporal context in which the pathway is engaged [34]. Selective manipulation of cAMP-PKA signaling restricted to a perinuclear compartment enhanced neurite extension in primary rat hippocampal neurons [35]. Similarly, in hippocampal HT22 cells, transmembrane and soluble adenylyl cyclase-derived cAMP pools have been shown to play distinct roles in corticotropin-releasing hormone receptor 1 signaling, and corticotropin-releasing hormone-induced neurite outgrowth depends preferentially on soluble adenylyl cyclase-derived cAMP [10]. In the present study, PGE_2_ and PGI_2_ induced comparable increases in whole-cell cAMP accumulation at early time points (10 min), and PGI_2_-treated cells maintained whole-cell cAMP levels comparable to or higher than those of PGE_2_-treated cells at 3 h. Despite this, CREB phosphorylation returned to baseline in PGI_2_-treated cells by 3 h, whereas it remained elevated in PGE_2_-treated cells. This dissociation indicates that the difference between PGE_2_ and PGI_2_ signaling cannot be explained simply by the magnitude of whole-cell cAMP accumulation. It suggests that EP2 and IP receptors may generate spatially or temporally distinct cAMP pools with differing capacities to sustain nuclear CREB activation. One potential receptor-level mechanism involves differences in post-endocytic trafficking. EP2 internalizes early endosomes and sustains cAMP production from this compartment [36], whereas IP undergoes classical endocytosis associated with receptor desensitization and degradation [37,38]. If such trafficking differences are preserved in NSC-34 cells, EP2-derived endosomal cAMP may preferentially support sustained nuclear CREB activation, whereas IP-derived cAMP may remain spatially restricted near the plasma membrane. However, this interpretation remains hypothetical, as the present study measured only whole-cell cAMP and did not assess compartment-resolved signaling or receptor localization. Further studies using compartment-specific cAMP sensors and analyses of receptor trafficking will be required to test these mechanisms.

CREB is a stimulus-responsive transcription factor that links second-messenger signaling to gene expression programs in neurons. Previous studies have shown that CREB-dependent transcription is governed not only by signal amplitude but also by stimulus duration [39,40]. Across multiple neuronal systems, sustained CREB phosphorylation is associated with neuronal differentiation and growth responses, whereas transient CREB activation is insufficient. For example, in hippocampal neurons, only stimuli that maintain Ser133 phosphorylation beyond a threshold duration induce CRE-dependent gene expression, while equally intense but transient signals do not [39,40]. In organotypic striatal cultures, D1 receptor stimulation produces region-dependent differences in the duration of CREB phosphorylation, and only the sustained response correlates with downstream Fos induction [41]. Similarly, in PC12 cells, sustained CREB activation is required for GPCR-mediated neuronal differentiation, whereas transient phosphorylation from the same pathway fails to support this process [42]. In motor neurons, sustained CREB activity is sufficient to overcome growth-inhibitory cues and promote axon regeneration in rat dorsal root ganglion after injury [43]. Consistent with this framework, the present study shows that PGE_2_ elicits more sustained CREB activation than PGI_2_, despite comparable early Gs-cAMP-PKA signaling. Although the p-CREB/total CREB ratio was numerically higher in PGI_2_-treated cells than that in PGE_2_-treated cells at early time points (10–30 min), PGI_2_ did not induce neurite outgrowth even at higher concentrations. These findings indicate that the peak CREB phosphorylation alone does not explain the neuritogenic response. Instead, neurite outgrowth in this model appears to be more closely associated with sustained CREB phosphorylation than with the magnitude of peak CREB activation. However, CREB phosphorylation was assessed only at a single PGI_2_ concentration (30 μM); different concentrations could alter the temporal profile of CREB activation. Furthermore, the present study did not include direct functional manipulation of CREB, precluding definitive conclusions about whether sustained CREB phosphorylation is necessary or sufficient for neurite outgrowth in this model.

Transcription factors such as CREB regulate neurite outgrowth partly by inducing downstream effector genes [40]. Follistatin is a secreted antagonist of TGF-β superfamily members, including bone morphogenetic proteins (BMPs) and activins [44]. The *Fst* promoter is regulated by CREB downstream of cAMP-PKA signaling in mouse gonadotrophs [45]. Follistatin has also been described as a developmentally regulated factor in neural differentiation and shown to promote neurite outgrowth in IMR-32 neuroblastoma cells [46]. BMP-2 inhibits neurite outgrowth and morphological differentiation in NSC-34 cells [47], and because follistatin can antagonize BMP-2 activity in other cellular contexts [44], its induction may relieve BMP-mediated suppression of neuritogenesis in NSC-34 cells. In this study, RNA sequencing at 4 h identified *Fst* as the only gene expressed at significantly higher levels in PGE_2_-treated cells than in PGI_2_-treated cells. The sustained CREB phosphorylation observed under PGE_2_ may contribute to the greater induction of *Fst* at this sampling point. However, several considerations limit this interpretation. Both PGs upregulated *Fst* relative to the control, suggesting that PGI_2_ can also induce *Fst* without sustained CREB phosphorylation. Moreover, the effect of follistatin is not universally pro-neuritogenic; in dorsal root ganglion cultures, it suppresses activin A-induced neurite outgrowth [48]. However, the functional role of follistatin in neurite outgrowth has not been directly evaluated in NSC-34 cells. Moreover, the relatively small number of differentially expressed genes in the PGE_2_ vs. PGI_2_ comparison may reflect the limitations of a single-time-point design with modest biological replication (*n* = 3 per group), potentially missing dynamic transcriptional changes relevant to neuritogenesis. Therefore, *Fst* should be considered a preliminary candidate, and its functional contribution to PGE_2_-induced neurite outgrowth, as well as whether its differential expression is driven by sustained CREB activation, remains to be determined.

NSC-34 cells retain several motor neuron-like properties [13,14] and provide a controlled system for comparing receptor-specific signaling within a shared cellular background. However, as a neuroblastoma/spinal cord hybrid line, they do not undergo uniform neuronal differentiation [49], and their physiological responses may diverge from those of primary motor neurons depending on the signaling context [50]. Therefore, the present findings should be interpreted as differences in signaling identified in a controlled motor neuron-like cell model. Validation in primary motor neuron cultures or human iPSC-derived motor neuron systems is warranted to determine the generalizability of these observations. Nevertheless, the present findings show that receptor identity within the Gs-coupled prostanoid family is closely associated with the neuritogenic outcomes within this model. Both IP and EP2 are functionally expressed and activate the Gs-cAMP-PKA cascade with comparable early kinetics and amplitude. However, only PGE_2_, whose neuritogenic effect is primarily mediated by EP2 signaling [15], produced sustained CREB phosphorylation and induced neurite outgrowth. The dissociation between bulk cAMP levels and sustained CREB activation is consistent with compartmentalized signaling; however, only whole-cell cAMP was measured, and compartment-specific cAMP dynamics were not directly assessed. To explore downstream transcriptional consequences of this divergence, we examined transcriptomic responses at 4 h. PGE_2_ and PGI_2_ elicited largely concordant responses, with *Fst* as the only gene significantly more highly expressed under PGE_2_ than under PGI_2_, while gene set enrichment analysis suggested distinct pathway-level enrichment patterns. Because RNA sequencing was performed at a single time point with limited biological replication, the analysis may not have captured dynamic changes in signaling and transcriptional responses. Therefore, *Fst* should be considered an exploratory candidate rather than a validated effector. Overall, the present findings support an association between sustained CREB phosphorylation and neuritogenic outcomes in this model system. However, further validation through targeted manipulation of CREB signaling and its downstream effectors is essential to establish causality.

## 5. Conclusions

In conclusion, although PGE_2_ and PGI_2_ elicited comparable early Gs-cAMP-PKA activation kinetics in NSC-34 motor neuron-like cells, only PGE_2_ induced neurite outgrowth. Sustained CREB phosphorylation was observed selectively under neuritogenic conditions and was associated with the PGE_2_ treatment. These findings suggest that differences in the temporal dynamics of CREB activation may contribute to distinct neuritogenic outcomes downstream of Gs-coupled prostanoid receptors. The dissociation between bulk cAMP accumulation and CREB phosphorylation kinetics further supports the possibility that receptor-specific compartmentalization of cAMP signaling may be involved. However, the underlying mechanisms remain to be elucidated. In addition, RNA sequencing identified *Fst* as the only gene significantly more highly expressed under PGE_2_ than under PGI_2_. Its potential contribution to the neuritogenic response remains unclear and warrants further investigation. This study should therefore be interpreted as a comparative analysis of signaling features rather than evidence of causal mechanisms.

## Data Availability

The RNA-seq raw data generated in this study have been deposited in the DNA Data Bank of Japan (DDBJ) Sequence Read Archive under accession numbers DRR914327–DRR914335 and are associated with BioProject PRJDB40580. The processed gene expression data and associated metadata have been deposited in the DDBJ Genomic Expression Archive (GEA) under accession number E-GEAD-1225. Further inquiries can be directed to the corresponding author.

## Supplementary Materials

The following supporting information can be downloaded at: https://www.mdpi.com/article/10.3390/cells15111004/s1. Table S1: Full list of differentially expressed genes identified in NSC-34 cells after PGE_2_ or PGI_2_ treatment.
