# Supplementary material for: Sustained CREB Phosphorylation Is Associated with Neuritogenic Prostanoid Signaling in NSC-34 Cells
Source: Cells. 2026 May 29;15(11):1004. doi: 10.3390/cells15111004 (PMC13257260; doi:10.3390/cells15111004)
Supplement: Supplementary file 1 [file cells-15-01004-s001.zip › cells-4312744-supplementary.pdf]

Table S1: Full list of differentially expressed genes identified in NSC-34 cells after PGE_2 or PGI_2 treatment.

comparison	treated_condition	gene_id	ensembl_core	symbol	gene_name	label	direction_in_treated	baseMean	log2FoldChange_original	log2FoldChange_shrunk	log2FoldChange_treated_aligned	lfcSE	lfcSE_shrunk	stat	pvalue	padj
Ctrl vs PGE_2	PGE_2	ENSMUSG00000021765.10	ENSMUSG00000021765	Fst	follistatin	Fst	up	1070.437397	-4.858822698	-4.813060907	4.813060907	0.26709811	0.266279516	-18.19115341	6.07E-74	2.52E-70
Ctrl vs PGE_2	PGE_2	ENSMUSG00000058624.14	ENSMUSG00000058624	Gda	guanine deaminase	Gda	up	55.78425677	-4.263299747	-4.107271106	4.107271106	0.515524175	0.512551925	-8.269834762	1.34E-16	1.05E-13
Ctrl vs PGE_2	PGE_2	ENSMUSG00000006311.9	ENSMUSG00000006311	Etv2	ets variant 2	Etv2	up	46.65453493	-3.868187103	-3.759623036	3.759623036	0.436514691	0.437504272	-8.861527881	7.89E-19	7.57E-16
Ctrl vs PGE_2	PGE_2	ENSMUSG00000087196.4	ENSMUSG00000087196	Gm13373	predicted gene 13373	Gm13373	up	52.09741079	-3.823066954	-3.706828863	3.706828863	0.449558318	0.452429385	-8.504051206	1.83E-17	1.63E-14
Ctrl vs PGE_2	PGE_2	ENSMUSG00000010064.16	ENSMUSG00000010064	Slc38a3	solute carrier family 38, member 3	Slc38a3	up	997.7831727	-2.160791658	-2.138595519	2.138595519	0.100631862	0.101558217	-21.47224166	2.83E-102	1.77E-98
Ctrl vs PGE_2	PGE_2	ENSMUSG00000026483.14	ENSMUSG00000026483	Niban1	niban apoptosis regulator 1	Niban1	up	994.9108578	-2.030993509	-1.938731931	1.938731931	0.196982379	0.200187074	-10.31053397	6.31E-25	7.88E-22
Ctrl vs PGE_2	PGE_2	ENSMUSG00000111926.3	ENSMUSG00000111926	Gm46204	predicted gene, 46204	Gm46204	up	76.87558256	-2.031110221	-1.830493253	1.830493253	0.291666453	0.29867987	-6.963811569	3.31E-12	1.59E-09
Ctrl vs PGE_2	PGE_2	ENSMUSG00000018648.16	ENSMUSG00000018648	Dusp14	dual specificity phosphatase 14	Dusp14	up	67.95945843	-2.074171498	-1.794638599	1.794638599	0.346075499	0.359824486	-5.993407524	2.05E-09	5.83E-07
Ctrl vs PGE_2	PGE_2	ENSMUSG00000033721.17	ENSMUSG00000033721	Vav3	vav 3 oncogene	Vav3	up	54.00738258	-2.128576352	-1.783128685	1.783128685	0.38489201	0.412761466	-5.530320958	3.20E-08	7.25E-06
Ctrl vs PGE_2	PGE_2	ENSMUSG00000034586.15	ENSMUSG00000034586	Hid1	HID1 domain containing	Hid1	up	3636.323288	-1.773985847	-1.760991972	1.760991972	0.071843091	0.071827985	-24.69250444	1.29E-134	1.61E-130
Ctrl vs PGE_2	PGE_2	ENSMUSG00000032548.15	ENSMUSG00000032548	Slco2a1	solute carrier organic anion transporter family, member 2a1	Slco2a1	up	310.3801226	-1.91793228	-1.704418533	1.704418533	0.298102375	0.301641847	-6.433804095	1.24E-10	5.18E-08
Ctrl vs PGE_2	PGE_2	ENSMUSG00000063889.17	ENSMUSG00000063889	Crem	cAMP responsive element modulator	Crem	up	640.3805439	-1.632624436	-1.554928719	1.554928719	0.178735096	0.177771897	-9.134324897	6.58E-20	7.46E-17
Ctrl vs PGE_2	PGE_2	ENSMUSG00000023868.18	ENSMUSG00000023868	Pde10a	phosphodiesterase 10A	Pde10a	up	1597.697871	-1.619597697	-1.453995568	1.453995568	0.261341965	0.261826738	-6.197235469	5.75E-10	1.84E-07
Ctrl vs PGE_2	PGE_2	ENSMUSG00000020044.14	ENSMUSG00000020044	Timp3	tissue inhibitor of metalloproteinase 3	Timp3	up	164.6846957	-1.746714871	-1.438012839	1.438012839	0.343857477	0.380190994	-5.079764112	3.78E-07	7.25E-05
Ctrl vs PGE_2	PGE_2	ENSMUSG00000051359.16	ENSMUSG00000051359	Ncald	neurocalcin delta	Ncald	up	132.5528627	-1.543143422	-1.387780484	1.387780484	0.25309217	0.254588961	-6.097159855	1.08E-09	3.21E-07
Ctrl vs PGE_2	PGE_2	ENSMUSG00000028341.10	ENSMUSG00000028341	Nr4a3	nuclear receptor subfamily 4, group A, member 3	Nr4a3	up	194.1274953	-1.444435701	-1.363798174	1.363798174	0.1840304	0.183712543	-7.848897257	4.20E-15	2.62E-12
Ctrl vs PGE_2	PGE_2	ENSMUSG00000092035.10	ENSMUSG00000092035	Peg10	paternally expressed 10	Peg10	up	14075.21489	-1.355850607	-1.321581494	1.321581494	0.12088095	0.120769251	-11.2164126	3.39E-29	6.04E-26
Ctrl vs PGE_2	PGE_2	ENSMUSG00000022296.11	ENSMUSG00000022296	Baalc	brain and acute leukemia, cytoplasmic	Baalc	up	106.357156	-1.481023688	-1.311679499	1.311679499	0.261666004	0.268901703	-5.659977464	1.51E-08	3.63E-06
Ctrl vs PGE_2	PGE_2	ENSMUSG00000025475.18	ENSMUSG00000025475	Adgra1	adhesion G protein-coupled receptor A1	Adgra1	up	354.2232314	-1.399636641	-1.293505287	1.293505287	0.210195794	0.211275542	-6.658728119	2.76E-11	1.25E-08
Ctrl vs PGE_2	PGE_2	ENSMUSG00000026721.17	ENSMUSG00000026721	Rabgap1l	RAB GTPase activating protein 1-like	Rabgap1l	up	1645.191264	-1.291177594	-1.267916869	1.267916869	0.099843272	0.099873619	-12.93204408	2.97E-38	6.17E-35
Ctrl vs PGE_2	PGE_2	ENSMUSG00000032135.16	ENSMUSG00000032135	Mcam	melanoma cell adhesion molecule	Mcam	up	1760.471705	-1.253013939	-1.231241307	1.231241307	0.09651786	0.09662626	-12.98219774	1.54E-38	3.85E-35
Ctrl vs PGE_2	PGE_2	ENSMUSG00000024500.21	ENSMUSG00000024500	Ppp2r2b	protein phosphatase 2, regulatory subunit B, beta	Ppp2r2b	up	634.380837	-1.258559001	-1.225764032	1.225764032	0.118110255	0.118357347	-10.6557978	1.64E-26	2.55E-23
Ctrl vs PGE_2	PGE_2	ENSMUSG00000037458.16	ENSMUSG00000037458	Azin1	antizyme inhibitor 1	Azin1	up	14625.71831	-1.275153922	-1.219479236	1.219479236	0.153046592	0.153733043	-8.331802112	7.96E-17	6.62E-14
Ctrl vs PGE_2	PGE_2	ENSMUSG00000000628.11	ENSMUSG00000000628	Hk2	hexokinase 2	Hk2	up	2874.916404	-1.189032427	-1.176162971	1.176162971	0.074045775	0.074154508	-16.05807248	5.02E-58	1.57E-54
Ctrl vs PGE_2	PGE_2	ENSMUSG00000028197.5	ENSMUSG00000028197	Col24a1	collagen, type XXIV, alpha 1	Col24a1	up	140.7920982	-1.303221008	-1.170004888	1.170004888	0.229461984	0.239221973	-5.679463699	1.35E-08	3.35E-06
Ctrl vs PGE_2	PGE_2	ENSMUSG00000026107.12	ENSMUSG00000026107	Nabp1	nucleic acid binding protein 1	Nabp1	up	1922.630126	-1.187211128	-1.136438812	1.136438812	0.144874683	0.146102127	-8.194745293	2.51E-16	1.74E-13
Ctrl vs PGE_2	PGE_2	ENSMUSG00000021587.6	ENSMUSG00000021587	Pcsk1	proprotein convertase subtilisin/kexin type 1	Pcsk1	up	43.20911057	-1.708405482	-1.117385726	1.117385726	0.396265167	0.573659795	-4.311268372	1.62E-05	0.001729958
Ctrl vs PGE_2	PGE_2	ENSMUSG00000032265.15	ENSMUSG00000032265	Tent5a	terminal nucleotidyltransferase 5A	Tent5a	up	592.5437608	-1.167092492	-1.116888066	1.116888066	0.143600474	0.144954093	-8.127358228	4.39E-16	2.88E-13
Ctrl vs PGE_2	PGE_2	ENSMUSG00000040624.19	ENSMUSG00000040624	Plekhg1	pleckstrin homology domain containing, family G (with RhoGef domain) member 1	Plekhg1	up	155.0204881	-1.303166022	-1.093504601	1.093504601	0.264921481	0.314244046	-4.919065142	8.70E-07	1.53E-04
Ctrl vs PGE_2	PGE_2	ENSMUSG00000050711.8	ENSMUSG00000050711	Scg2	secretogranin II	Scg2	up	996.3749884	-1.160410217	-1.071285303	1.071285303	0.187104299	0.192638212	-6.2019431	5.58E-10	1.83E-07
Ctrl vs PGE_2	PGE_2	ENSMUSG00000027297.16	ENSMUSG00000027297	Ltk	leukocyte tyrosine kinase	Ltk	down	608.1550423	1.093874766	1.057520671	-1.057520671	0.121049429	0.122151375	9.036595864	1.62E-19	1.68E-16
Ctrl vs PGE_2	PGE_2	ENSMUSG00000026387.16	ENSMUSG00000026387	Sctr	secretin receptor	Sctr	up	67.19833389	-1.441170055	-1.050407576	1.050407576	0.325426939	0.45116055	-4.428551789	9.49E-06	0.001137959
Ctrl vs PGE_2	PGE_2	ENSMUSG00000025582.5	ENSMUSG00000025582	Nptx1	neuronal pentraxin 1	Nptx1	up	312.0205202	-1.189789379	-1.048223867	1.048223867	0.224636087	0.248878004	-5.296519338	1.18E-07	2.54E-05
Ctrl vs PGE_2	PGE_2	ENSMUSG00000023800.16	ENSMUSG00000023800	Tiam2	T cell lymphoma invasion and metastasis 2	Tiam2	up	1138.391313	-1.117421079	-1.031763113	1.031763113	0.181489286	0.187825397	-6.156953427	7.42E-10	2.31E-07
Ctrl vs PGE_2	PGE_2	ENSMUSG00000042770.9	ENSMUSG00000042770	Hebp1	heme binding protein 1	Hebp1	up	1006.604776	-1.195574207	-1.031487014	1.031487014	0.235065261	0.271458034	-5.086137358	3.65E-07	7.12E-05
Ctrl vs PGE_2	PGE_2	ENSMUSG00000039385.6	ENSMUSG00000039385	Cdh6	cadherin 6	Cdh6	up	442.0633663	-2.17259799	-1.00662879	1.00662879	0.532615989	0.807953238	-4.079107719	4.52E-05	0.004104867
Ctrl vs PGI_2	PGI_2	ENSMUSG00000058624.14	ENSMUSG00000058624	Gda	guanine deaminase	Gda	up	55.78425677	-3.750995399	-3.431800714	3.431800714	0.517312074	0.496852715	-7.25093341	4.14E-13	5.25E-10
Ctrl vs PGI_2	PGI_2	ENSMUSG00000021765.10	ENSMUSG00000021765	Fst	follistatin	Fst	up	1070.437397	-3.484032975	-3.399676666	3.399676666	0.267861753	0.264631755	-13.00683256	1.12E-38	4.32E-35
Ctrl vs PGI_2	PGI_2	ENSMUSG00000087196.4	ENSMUSG00000087196	Gm13373	predicted gene 13373	Gm13373	up	52.09741079	-3.208427227	-2.990686158	2.990686158	0.452535586	0.442970047	-7.089889334	1.34E-12	1.38E-09
Ctrl vs PGI_2	PGI_2	ENSMUSG00000006311.9	ENSMUSG00000006311	Etv2	ets variant 2	Etv2	up	46.65453493	-2.759536846	-2.55321796	2.55321796	0.444490462	0.454240149	-6.208315099	5.36E-10	4.60E-07
Ctrl vs PGI_2	PGI_2	ENSMUSG00000010064.16	ENSMUSG00000010064	Slc38a3	solute carrier family 38, member 3	Slc38a3	up	997.7831727	-2.155162217	-2.14521772	2.14521772	0.100284288	0.100651654	-21.49052725	1.91E-102	1.48E-98
Ctrl vs PGI_2	PGI_2	ENSMUSG00000033721.17	ENSMUSG00000033721	Vav3	vav 3 oncogene	Vav3	up	54.00738258	-2.091628539	-1.867306164	1.867306164	0.383471811	0.432085455	-5.45445188	4.91E-08	3.45E-05
Ctrl vs PGI_2	PGI_2	ENSMUSG00000026483.14	ENSMUSG00000026483	Niban1	niban apoptosis regulator 1	Niban1	up	994.9108578	-1.849577764	-1.789759358	1.789759358	0.196962897	0.204670484	-9.39048822	5.97E-21	1.15E-17
Ctrl vs PGI_2	PGI_2	ENSMUSG00000034586.15	ENSMUSG00000034586	Hid1	HID1 domain containing	Hid1	up	3636.323288	-1.605761155	-1.595682861	1.595682861	0.071818827	0.07225489	-22.35849873	9.98E-111	1.54E-106
Ctrl vs PGI_2	PGI_2	ENSMUSG00000032548.15	ENSMUSG00000032548	Slco2a1	solute carrier organic anion transporter family, member 2a1	Slco2a1	up	310.3801226	-1.614653159	-1.422616023	1.422616023	0.298301317	0.321455385	-5.412826116	6.20E-08	4.17E-05
Ctrl vs PGI_2	PGI_2	ENSMUSG00000032135.16	ENSMUSG00000032135	Mcam	melanoma cell adhesion molecule	Mcam	up	1760.471705	-1.300033986	-1.277025804	1.277025804	0.096235628	0.096609515	-13.50886368	1.39E-41	7.14E-38
Ctrl vs PGI_2	PGI_2	ENSMUSG00000111926.3	ENSMUSG00000111926	Gm46204	predicted gene, 46204	Gm46204	up	76.87558256	-1.464035258	-1.249110839	1.249110839	0.294667139	0.332981169	-4.968437488	6.75E-07	3.26E-04
Ctrl vs PGI_2	PGI_2	ENSMUSG00000028197.5	ENSMUSG00000028197	Col24a1	collagen, type XXIV, alpha 1	Col24a1	up	140.7920982	-1.366753624	-1.242656266	1.242656266	0.22788459	0.231928517	-5.997569312	2.00E-09	1.63E-06
Ctrl vs PGI_2	PGI_2	ENSMUSG00000024500.21	ENSMUSG00000024500	Ppp2r2b	protein phosphatase 2, regulatory subunit B, beta	Ppp2r2b	up	634.380837	-1.212014128	-1.177045619	1.177045619	0.11770789	0.117904445	-10.29679599	7.28E-25	2.25E-21
Ctrl vs PGI_2	PGI_2	ENSMUSG00000092035.10	ENSMUSG00000092035	Peg10	paternally expressed 10	Peg10	up	14075.21489	-1.190367965	-1.153463987	1.153463987	0.120876765	0.120969874	-9.847781414	7.01E-23	1.81E-19
Ctrl vs PGI_2	PGI_2	ENSMUSG00000051359.16	ENSMUSG00000051359	Ncald	neurocalcin delta	Ncald	up	132.5528627	-1.308285271	-1.149397529	1.149397529	0.253271358	0.265768536	-5.16554766	2.40E-07	1.48E-04
Ctrl vs PGI_2	PGI_2	ENSMUSG00000018648.16	ENSMUSG00000018648	Dusp14	dual specificity phosphatase 14	Dusp14	up	67.95945843	-1.548996271	-1.135698321	1.135698321	0.348589534	0.528394116	-4.443610952	8.85E-06	0.003038358
Ctrl vs PGI_2	PGI_2	ENSMUSG00000023868.18	ENSMUSG00000023868	Pde10a	phosphodiesterase 10A	Pde10a	up	1597.697871	-1.236962109	-1.043300295	1.043300295	0.261423781	0.310586827	-4.731635758	2.23E-06	9.56E-04
Ctrl vs PGI_2	PGI_2	ENSMUSG00000025582.5	ENSMUSG00000025582	Nptx1	neuronal pentraxin 1	Nptx1	up	312.0205202	-1.133970079	-1.006173599	1.006173599	0.224228485	0.235092263	-5.057207959	4.25E-07	2.27E-04
PGE_2 vs PGI_2	PGE_2	ENSMUSG00000021765.10	ENSMUSG00000021765	Fst	follistatin	Fst	up	1070.437397	1.374789723	1.04411991	1.04411991	0.251228472	0.307531131	5.472268773	4.44E-08	2.40E-04
